# Estimates of DNA strand breakage in bottlenose dolphin (*Tursiops truncatus*) leukocytes measured with the Comet and DNA diffusion assays

**DOI:** 10.1590/S1415-47572009005000030

**Published:** 2009-03-06

**Authors:** Adriana Díaz, Sandra Carro, Livia Santiago, Juan Estévez, Celia Guevara, Miriam Blanco, Laima Sánchez, Liena Sánchez, Nirka López, Danilo Cruz, Ronar López, Elizabeth B. Cuetara, Jorge Luis Fuentes

**Affiliations:** 1 Departamento de Radiobiología, Centro de Aplicaciones Tecnológicas y Desarrollo Nuclear, C. HabanaCuba; 2 Departamento de Salud Animal, Acuario Nacional de Cuba, C. HabanaCuba; 3 Laboratorio de Farmacología Clínica y Experimental, Instituto Nacional de Oncología y Radiobiología, C. HabanaCuba; 4 Laboratorio de Microbiología y Mutagénesis Ambiental, Escuela de Biología, Facultad de Ciencias, Universidad Industrial de Santander, BucaramangaColombia

**Keywords:** Comet assay, DNA diffusion assay, DNA strand breakage, *Tursiops truncatus*

## Abstract

The analysis of DNA damage by mean of Comet or single cell gel electrophoresis (SCGE) assay has been commonly used to assess genotoxic impact in aquatic animals being able to detect exposure to low concentrations of contaminants in a wide range of species. The aims of this work were 1) to evaluate the usefulness of the Comet to detect DNA strand breakage in dolphin leukocytes, 2) to use the DNA diffusion assay to determine the amount of DNA strand breakage associated with apoptosis or necrosis, and 3) to determine the proportion of DNA strand breakage that was unrelated to apoptosis and necrosis. Significant intra-individual variation was observed in all of the estimates of DNA damage. DNA strand breakage was overestimated because a considerable amount (~29%) of the DNA damage was derived from apoptosis and necrosis. The remaining DNA damage in dolphin leukocytes was caused by factors unrelated to apoptosis and necrosis. These results indicate that the DNA diffusion assay is a complementary tool that can be used together with the Comet assay to assess DNA damage in bottlenose dolphins.

## Introduction

The single cell gel electrophoresis (SCGE) or Comet assay, as introduced by Singh *et al.* (1988), is a technique that detects DNA strand breakage and alkali labile sites by measuring the migration of DNA from immobilized individual cell nuclei. In this assay, cells are embedded in agarose gel on microscopic slides and lysed and electrophoresed under alkaline condition. Cells with damaged DNA show increased migration of DNA fragments from the nucleus. The length of the migration indicates the amount of DNA breakage, and the DNA damage can be estimated by both manual microscopic and computerized image scoring analyses (Olive *et al.*, 1990; McKelvey-Martin *et al.*, 1993; Fairbairn *et al.*, 1995; Kobayashi *et al.*, 1995). The minimal technical requirements for conducting this assay in human cells *in vitro* and *in vivo* have been well established (Tice *et al.*, 2000; Hartmann *et al.*, 2003).

The widespread use of the Comet assay in biomonitoring studies of aquatic organisms is related mainly to its simplicity, low cost and greater sensitivity to xenobiotics when compared with other techniques (Mitchelmore and Chipman, 1998; Cotelle and Ferard, 1999; Lee and Steinert, 2003; Frenzilli *et al.*, 2009). The Comet assay has been used to detect DNA damage induced by hydrogen peroxide and methyl mercury in bottlenose dolphin (*Tursiops truncatus*) lymphocytes *in vitro* (Betti and Nigro, 1996; Taddei *et al.*, 2001), and by hydrogen peroxide and benzo[a]pyrene-7,8-dihydro-diol-9,10-epoxide in sea lion lymphocytes (El-Zein *et al.*, 2006). These results suggest that this assay may be a sensitive tool for monitoring DNA damage in marine mammals.

However, the use of the Comet assay in biomonitoring studies has been questioned because positive results in this assay do not necessarily reflect genotoxicity but may arise from DNA damage, *i.e.*, double-strand breakage, associated with apoptosis and necrosis (Olive *et al.*, 1993; Olive and Banath, 1995; Steinert, 1996; Godard *et al.*, 1999; Tice *et al.*, 2000; Wada *et al.*, 2003). To overcome this limitation, the Comet assay may be used in combination with related methodologies that estimate DNA fragmentation associated with apoptosis and necrosis, such as the DNA diffusion assay (Singh, 2000a,b). In the latter assay, small molecular weight DNA fragments generated during apoptosis and necrosis diffuse in the agarose matrix to give an apparent nuclear diameter that is ~3 times greater than the mean nuclear size as a consequence of the high dispersion of DNA. Based on the structural differences between apoptotic and necrotic nuclei, Singh (2000a,b) recommended that the DNA diffusion assay be used to distinguish apoptotic from necrotic cells. However, other investigators have been unable to differentiate between apoptotic and necrotic cells when using this assay (Gichner *et al.*, 2005). The use of the DNA diffusion assay to assess apoptosis has been reviewed by Singh (2005) and its application in a small number of environmental studies has yielded promising results (Nigro *et al.*, 2002; Frenzilli *et al.*, 2004; Del Barga *et al.*, 2006).

We have initiated a long-term monitoring project aimed at evaluating the life quality of bottlenose dolphins living in semi-captive and captive conditions. In view of the potential use of the Comet assay in the biomonitoring of dolphins and that a concurrent assessment of apoptosis, necrosis and genotoxin-induced DNA strand breakage is critical for the correction interpretation of this assay, the aims of this work were: 1) to evaluate the usefulness of the Comet or single cell gel electrophoresis (SCGE) assay to detect DNA strand breakage in dolphin leukocytes, 2) to use the DNA diffusion assay to determine the amount of DNA strand breakage associated with apoptosis and necrosis, and 3) to determine the proportion of DNA strand breakage that was unrelated to apoptosis and necrosis.

**Figure 1:**
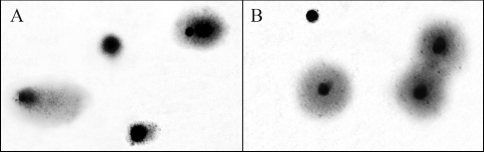
A. Bottlenose dolphin leukocytes analyzed by the Comet assay. Nuclei from undamaged leukocytes consisted of a head (nuclear core) with little or no DNA migrating into the tail region. Nuclei from damaged leukocytes consisted of a head with DNA migrating into the tail region as a result of strand breakage. B. Bottlenose dolphin leukocytes analyzed by the DNA diffusion assay. Nuclei that diffused through the agarose gel were considered as apoptotic/necrotic nuclei.

## Materials and Methods

### Capture and living conditions of the dolphins

Twenty-five bottlenose dolphins (Table 1) were captured in the Sabana-Camagüey archipelago on the northern coast of Cuba and their sex was determined visually. Age was estimated based on the dolphins size, teething state and body marks at the time of capture. The dolphins were initially quarantined in sea-hoops located close to the capture site where they were examined by a veterinarian and underwent blood and spiracle analyses. Only visually healthy and asymptomatic dolphins that willingly consumed frozen fish were transferred to the dolphinarium at the Cuban National Aquarium (CNA). The dolphinarium was supplied with water from a subterranean well via a semi-closed feeding system with filters and a water renewal rate of 10% per hour. This system was permanently and automatically supplemented with sodium hypochlorite (final concentration of free chloride: 0.3 mg/L). The quality of water used in the dolphinarium was based on the parameters established by the Cuban guidelines for fishery zones (NC25:1999).

### Blood sampling

Blood samples were obtained by vacuum using sterile, heparinized tubes and were always shipped and stored on ice until assayed.

### Analysis of serum genotoxicity

Serum genotoxicity was measured indirectly using the SOS Chromotest (Quillardet *et al.*, 1982) with modifications. *Escherichia coli* PQ37 cells were grown to an OD_600nm_ of 0.4 in Luria-Bertani (LB) media supplemented with ampicillin (50 μg/mL) at 37 °C, with shaking (100 rpm). Exponential phase cultures were diluted ten-fold in fresh 2X LB media supplemented with ampicillin (100 μg/mL) and then dispensed in Eppendorf tubes (250 μL per tube) containing 225 μL of serum. When metabolic activation was required, 25 μL of phenobarbital/5,6 benzoflavone-induced rat liver S9 fraction from Moltox was used (final concentration in the activation mixture: 0.4%, v/v). In experiments without metabolic activation, the rat liver S9 fraction was substituted with sterile distilled water. The reference mutagens 2-acethyl-aminofluorene (500 μg/mL) and γ-rays (150 Gy delivered by a Co^60^ PX-γ-30M Russian irradiator at a dose rate of 33-42 Gy/min) were used as positive controls in experiments with and without metabolic activation, respectively. The cells were exposed to serum samples for 30 min at 8 °C and then cultured for 2 h at 37 °C. β-galactosidase and alkaline phosphatase activities were assayed as described by Fuentes *et al.*, (2006). The criterion for genotoxicity was the SOS induction factor (SOSIF), as defined by Quillardet *et al.* (1989): SOSIF = [β-galactosidase/alkaline phosphatase]_treatment_/[β-galactosidase/alkaline phosphatase]_negative__control_. Serum samples were classified as not genotoxic (SOSIF < 1.5), inconclusive (SOSIF = 1.5-2.0) or genotoxic (SOSIF > 2.0) (Kevekordes *et al.*, 1999).

### Estimation of DNA strand breakage in dolphin leukocytes

DNA strand breakage in dolphin leukocytes was estimated by using the Comet assay, as described by Singh *et al.* (1988), with modifications to the silver staining as indicated by Garcia *et al.* (2004). The DNA damage was scored (see Figure 1) based on five categories (0-4), as indicated by Collins *et al.* (1997). The total amount of DNA strand breakage was expressed in total arbitrary units (AU_T_) as follows: AU_T_ = N_0_ x 0 + N_1_ x 1 + N_2_ x 2 + N_3_ x 3 + N_4_ x 4, where N_i_ is the number of nuclei scored in each category (Collins, 2002). One hundred cells per slide and two slides per blood sample were analyzed and the results of at least two independent experiments were averaged to obtain the AU_T_ for each dolphin.

Since positive Comet results do not necessarily reflect genotoxicity because DNA strand breakage may be associated with cellular apoptosis and necrosis, we used the DNA diffusion assay to determine the percentage of apoptotic/necrotic cells in each blood sample. For this assay, the cells were processed in a manner similar to the Comet assay, except that the nuclei were not subjected to electrophoresis. Nuclei with a diameter > 3 times the mean nuclear diameter were considered apoptotic/necrotic (Nigro *et al.*, 2002). The total number of nuclei (minimum of 100 cells per slide) and the number of apoptotic/necrotic nuclei in each field were counted and the latter then expressed as a percentage of the former. As in the Comet assay, two slides per blood sample were analyzed and the results of at least two independent experiments were averaged to obtain the percentage of apoptotic/necrotic nuclei for each dolphin.

Based on the total number of DNA strand breakages (AU_T_) estimated with the Comet assay and the percentage of apoptotic/necrotic nuclei (%N_apoptotic/necrotic_) for each dolphin, the proportion of remaining DNA strand breakages was calculated (in arbitrary units) as:

$${\mathrm{AU}}_{R}={\mathrm{AU}}_{T}-\frac{\%N_{\mathrm{apoptotic/necrotic}}\times {\mathrm{AU}}_{T}}{100}$$

where AU_R_ corresponds to non-apoptotic/necrotic DNA strand breakages.

### Statistical analysis

The results were expressed as the mean ± S.E.M., where appropriate. In all cases, the data passed the Kolmogorov-Smirnov and F-maximum tests for normality and variance homogeneity, respectively, so that parametric tests were used to analyze the data. When a significant F-value was obtained in one-way analysis of variance (ANOVA) the groups were subsequently compared with Students *t*-test. Product-moment (Pearson) correlation analysis was used to examine the relationship between estimates of DNA damage (AU_T_, %N_apoptotic/necrotic_ and AU_R_). A value of p < 0.05 indicated significance. All statistical analyses were done with STATISTICA V.6 software (StatSoft Inc).

## Results

Table 2 shows the total DNA strand breakage in dolphin leukocytes, expressed as total arbitrary units (AU_T_) of the Comet assay. There was significant inter-individual variation (p < 0.05 in ANOVA) in the proportion of DNA strand breakage between the dolphins Jade (93 ± 48) and Mara (305 ± 28) (mean: 191 ± 34). Table 2 also shows the percentage of apoptotic/necrotic nuclei (%N_apoptotic/necrotic_) for each dolphin; there was significant inter-individual variation (p < 0.05 in ANOVA) between the dolphins Moon (6 ± 0) and Maja (77 ± 0) (mean: 29 ± 4).

The proportion of DNA strand breakage (AU_R_) not attributable to apoptosis and necrosis was calculated based on the total DNA strand breakage (AU_T_) estimated with the Comet assay and the percentages of apoptotic/necrotic nuclei (%N_apoptotic/necrotic_). The AU_R_ ranged from 34 ± 1 for Maja to 238 ± 33 for Mihai (mean: 125 ± 12). The AU_R_ estimates, but not the AU_T_, were significantly higher (*t*-value = - 2.16, p < 0.03) in males than females (164 ± 30 *vs.* 119 ± 14, respectively), suggesting that AU_R_ is a better indicator than AU_T_ for detecting DNA strand breakage in bottlenose dolphins. This finding contrasts with the results reported for beluga whales using chromosomal aberration and sister chromatid exchange assays (Gauthier *et al.*, 1999), but is not surprising since chromosomal aberrations occur on a much different scale than strand breakage and may be attributed to different factors. Both of the estimates of DNA damage (AU_T_ and AU_R_) increased significantly with dolphin age (data not shown), in agreement with previous findings obtained using the micronucleus assay (Zamora-Perez *et al.*, 2006).

To determine whether the residual (non-apoptotic/necrotic) DNA strand breakage (AU_R_) originated from exposure to genotoxic compounds we examined the genotoxicity of dolphin serum using the SOS chromotest (Quillardet *et al.*, 1982). This test detects a wide range of genotoxic compounds, including marine contaminants such as polycyclic aromatic hydrocarbons and organochlorine compounds (Quillardet and Hofnung, 1993). In experiments with metabolic activation, the SOSIF values ranged from 0.2 ± 0.0 to 0.8 ± 0.0 (mean: 0.5 ± 0.2) while in experiments without metabolic activation this indicator ranged from 0.3 ± 0.0 to 0.9 ± 0.6 (mean: 0.6 ± 0.4) (Table 2). These data indicated that there were no genotoxic compounds in dolphin blood.

## Discussion

In this work, we combined the Comet and DNA diffusion assays to measure DNA strand breakage in peripheral blood leukocytes of bottlenose dolphins. Nearly a third (~29%) of the DNA strand breakages arose from apoptotic/necrotic events and led to overestimation of DNA cleavage by the Comet assay, as also previously observed for mussels (Steinert, 1996), humans (Tice *et al.*, 2000) and sea lions (El-Zein *et al.*, 2006). The total DNA strand breakage (AU_T_) in dolphin leukocytes was 183 ± 17 AU, indicating a moderate level of DNA cleavage. However, as indicated above, this value was overestimated because of the influence of apoptotic/necrotic events. We therefore computed the value for residual (non-apoptotic/necrotic) DNA strand breakage (AU_R_ = 125 ± 12) and found this to be negatively correlated (r = -0.31, p < 0.05) with the percentage of apoptotic/necrotic nuclei. Hence, this parameter may provide a better estimate of non-apoptotic/necrotic DNA strand breakage. The mean AU_R_ corresponded to ~23% of DNA in the tail and was slightly higher than the limit for non-damaged nuclei (20% of DNA in the tail), as indicated by Collins *et al.* (1997).

Several studies have shown that marine contaminants such as heavy metals may induce apoptosis (Steinert, 1996; Shenker *et al.*, 2000; Waalkes *et al.*, 2000). We have investigated the involvement of Fe, Cu and Zn in DNA strand breakage induced by apoptotic/necrotic events and found a weak but significant correlation between serum copper levels and apoptotic/necrotic DNA strand breakage (data not shown). Thus, although the copper content of the dolphinarium water and dolphin serum was consistently low, the apoptosis observed here may have been induced by copper ions; no such relationship was observed for iron or zinc. Other environmental contaminants such as organochlorines and polycyclic aromatic hydrocarbons may also induce apoptosis (Salas and Burchiel, 1998; Shin *et al.*, 2000; Frenzilli *et al.*, 2004), but we have not detected genotoxic activity in dolphin serum using the SOS chromotest. Future studies measuring copper and other genotoxin levels in the environments where the dolphins used in this study normally live should improve our knowledge of the importance of this metal in causing apoptosis-related DNA damage.

The AU_R_ values clearly indicated that factors other than apoptosis/necrosis affect the integrity of dolphin leukocyte DNA. Based on the negative SOS chromotest results (Table 2) and the classification for DNA strand breakage in which a score of 0-100 indicated no DNA breakage or damage, 101-200 indicated little DNA damage, 201-300 indicated moderate DNA damage, and 301-400 indicated severe DNA damage, we expected baseline AU_R_ values of 0-100 AU, *i.e.*, no DNA damage (Kobayashi *et al.*, 1995; Collins *et al.*, 1997). However, nearly 78% of the dolphins had higher than expected AU_R_ values. Using the Comet assay, Taddei *et al.* (2001) estimated that DNA strand breakage in the nuclei of undamaged lymphocytes from bottlenose dolphins resulted in 16%-20% of their total DNA in the tail, which corresponded to 80-100 AU (Collins *et al.*, 1997). These studies suggest that there may be differences in the baseline estimates of DNA strand breakage obtained *in vitro* and *in vivo*, and that additional factors that affect the estimates of DNA cleavage must be considered during risk assessment studies *in vivo*. Variation in the extent of DNA strand breakage *in vivo* may reflect physiological conditions, such as a transient increase in oxidative stress caused by the diet or a sub-clinical infection (Collins *et al.*, 1997). An understanding of the influence of such factors on DNA cleavage in bottlenose dolphins should improve our estimates of baseline values for DNA strand breakage measured with the Comet assay.

## Conclusions

To our knowledge, this is the first estimate of DNA strand breakage obtained with the Comet assay in peripheral blood leukocytes of bottlenose dolphins. Our results indicate that this assay is sufficiently sensitive for assessing the influence of genotoxic substances in bottlenose dolphins. However, the Comet assay overestimates the extent of DNA strand breakage in these cells because of DNA cleavage caused by apoptotic and necrosis. In addition to apoptosis and necrosis, factors other than exposure to genotoxins may also affect the intactness of bottlenose dolphin DNA. Finally, our results indicate that the DNA diffusion assay is a suitable complementary tool for use alongside the Comet assay during risk assessment studies in bottlenose dolphins.

## Figures and Tables

**Table 1:** Life history data for the bottlenose dolphins used in this work.

N.	Dolphin name	Capture date	Origin	Sex^1^	Length^1^ (m)	Age group^1,2^
1	Ciceron	11/08/2000	Aguada key	M	2.13	Juvenile
2	Serena	12/08/2000	Aguada key	F	2.25	Adult
3	Salome	14/08/2000	Horseshoe key	F	2.19	Juvenile
4	Jade	04/12/2001	San Juan Point	F	2.08	Juvenile
5	Javy	04/12/2001	San Juan Point	F	1.98	Calf
6	Lía	15/07/2002	Santa Maria key	F	1.81	Calf
7	Lili	28/08/2002	Glad Point	F	2.04	Juvenile
8	Xena	25/10/2002	Caibarien bay	F	2.18	Juvenile
9	Maria	18/06/2003	Santa Maria key	F	2.49	Adult
10	Mara	18/06/2003	Santa Maria key	F	2.01	Juvenile
11	Merlin	18/06/2003	Santa Maria key	M	2.12	Juvenile
12	Maida	22/06/2003	Glad Point	F	2.40	Adult
13	Mihai	22/06/2003	Glad Point	M	1.86	Calf
14	Musa	22/06/2003	Glad Point	F	1.94	Calf
15	Marcelo	29/07/2003	Guarana key	M	2.40	Adult
16	Malù	08/08/2003	Horseshoe key	F	2.33	Adult
17	Monica	08/08/2003	Horseshoe key	F	2.28	Adult
18	Margarita	09/08/2003	Guarana key	F	2.07	Juvenile
19	Montse	02/09/2003	Guarana key	F	2.00	Juvenile
20	Melany	03/09/2003	Drunk key	F	2.28	Adult
21	Milo	08/09/2003	Guarana key	F	2.28	Adult
22	Moon	08/09/2003	Guarana key	F	2.19	Juvenile
23	Maja	08/09/2003	Guarana key	F	1.93	Calf
24	Mègano	18/10/2003	Guarana key	M	2.17	Juvenile
25	Milano	18/10/2003	Guarana key	M	2.11	Juvenile

^1^Determined at the time of capture. ^2^Age categories were: calf < 2.0 m long, juvenile 2.00-2.20 m long and adult > 2.21 m long.

**Table 2:** Leukocyte DNA damage and serum genotoxicity in bottlenose dolphins.

	Leukocyte DNA damage				Serum genotoxicity	
	AU_T_	% Nuclei_Ap/N_	AU_R_		SOSIF (S_9_ +)	SOSIF (S_9_ -)
Dolphin name						
Ciceron	257 ± 27	55 ± 14	117 ± 41		0.5 ± 0.0	0.7 ± 0.2
Serena	263 ± 93	26 ± 5	196 ± 72		0.4 ± 0.1	0.8 ± 0.1
Salome	242 ± 118	30 ± 8	166 ± 58		0.5 ± 0.0	0.7 ± 0.2
Jade	93 ± 48	26 ± 3	68 ± 33		0.4 ± 0.0	0.9 ± 0.3
Javy	154 ± 61	22 ± 2	120 ± 47		0.5 ± 0.2	0.7 ± 0.2
Lía	161 ± 73	28 ± 18	110 ± 43		0.6 ± 0.1	0.8 ± 0.3
Lili	160 ± 57	24 ± 5	118 ± 37		0.4 ± 0.1	0.5 ± 0.2
Xena	149 ± 56	29 ± 6	100 ± 29		0.6 ± 0.2	0.7 ± 0.1
Maria	186 ± 43	28 ± 16	131 ± 18		0.6 ± 0.0	0.4 ± 0.0
Mara	305 ± 28	64 ± 0	110 ± 10		0.2 ± 0.0	0.4 ± 0.0
Merlin	202 ± 62	29 ± 0	143 ± 44		0.8 ± 0.0	0.4 ± 0.0
Maida	102^†^	46^†^	55^†^		0.8^†^	0.8^†^
Mihai	298 ± 42	20 ± 0	238 ± 33		0.3 ± 0.0	0.5 ± 0.0
Musa	151^†^	28^†^	109^†^		0.2^†^	0.6^†^
Marcelo	205 ± 5	ND	ND		0.5 ± 0.0	0.4 ± 0.0
Malù	240 ± 22	50 ± 0	120 ± 11		0.6 ± 0.0	0.6 ± 0.0
Monica	241^†^	48^†^	125^†^		0.8^†^	0.4^†^
Margarita	239 ± 25	73 ± 0	65 ± 7		0.6 ± 0.0	0.5 ± 0.0
Montse	235 ± 47	20 ± 0	188 ± 37		0.2 ± 0.0	0.3 ± 0.0
Melany	180 ± 17	13 ± 0	157 ± 10		0.4 ± 0.0	0.5 ± 0.1
Milo	147 ± 75	23 ± 0	113 ± 58		0.4 ± 0.0	0.6 ± 0.1
Moon	217 ± 38	6 ± 0	204 ± 36		0.5 ± 0.0	0.4 ± 0.0
Maja	147 ± 7	77 ± 0	34 ± 1		0.3 ± 0.0	0.3 ± 0.0
Mègano	165 ± 119	ND	ND		0.3 ± 0.0	0.4 ± 0.0
Milano	206 ± 31	10 ± 0	186 ± 28		0.5 ± 0.1	0.5 ± 0.1
Mean	191 ± 34	29 ± 4	125 ± 12		0.5 ± 0.1	0.6 ± 0.2

The values are the mean±S.E.M., where appropriate. AU corresponds to arbitrary units, where AU_T_ is the total DNA damage as measured with the Comet assay, AU_R_ is the remaining non-apoptotic/necrotic DNA damage and %N_Ap/N_ is the percentage of apoptotic/necrotic nuclei. (†) Only one measurement was done. ND – Not determined. SOSIF – SOS induction factor in *Escherichia coli* PQ37 cells. The SOSIF values for the reference mutagens were 10.6 ± 2.2 for 500 μg of 2-acethyl-aminofluorene/mL (with metabolic activation) and 9.4 ± 0.4 for 150 Gy of γ-rays (without metabolic activation). The SOSIF for distilled water was 1.0 ± 0.0.
